# A prognostic index based on a fourteen long non-coding RNA signature to predict the recurrence-free survival for muscle-invasive bladder cancer patients

**DOI:** 10.1186/s12911-020-1115-2

**Published:** 2020-07-09

**Authors:** Xiaolong Zhang, Meng Zhang, Xuanping Zhang, Xiaoyan Zhu, Jiayin Wang

**Affiliations:** 1 School of Computer Science and Technology, Shaanxi Engineering Research Center of Medical and Health Big Data, Xi’an Jiaotong University, Xi’an, China; 2 State Key Laboratory of Oncology in South China, Collaborative Innovation Center for Cancer Medicine, Sun Yat-sen University Cancer Center, Guangzhou, China; 3 School of Medicine, Shenzhen University, Shenzhen, China; 4 Department of Urology, The First Affiliated Hospital of Anhui Medical University, Hefei, China

**Keywords:** Clinical decision-supporting, Muscle-invasive bladder cancer, Long non-coding RNA, Prognosis signature

## Abstract

**Background:**

Bladder cancer (BC) is regarded as one of the most fatal cancer around the world. Nevertheless, there still lack of sufficient markers to predict the prognosis of BC patients. Herein, we aim to establish a prognosis predicting signature based on long-noncoding RNA (lncRNA) for the invasive BC patients.

**Methods:**

The lncRNA expression profile was downloaded from The Cancer Genome Atlas (TCGA) database, along with the correlated clinicopathological information. The univariate Cox regression test was employed to screen out the recurrence-free survival (RFS)-related lncRNAs. Then, the LASSO method was conducted to construct the signature based on these RFS-related lncRNA candidates. Genes correlated with these fourteen lncRNAs were extracted from the mRNA expression profile, with the Pearson correlation coefficient > 0.60 or < − 0.40. Subsequently, the Proteomap pathway enrichment analyses were conducted to classify the function of these correlated genes. Furthermore, the multivariate analyses were executed to reveal the independent role of the proposed signature with the clinicopathological features.

**Results:**

We established an lncRNA-based RFS predicting signature by the LASSO Cox regression test, and proved its usage and stability on both the training and validation cohorts by the Kaplan-Meier and receiver operating characteristic (ROC) curves. Notably, the multivariate Cox regression analysis found that our classifier was an independent indicator for muscle-invasive BC patients rather than sex, age and tumor grade, with higher predictive value than the existing ones. Besides, we did the pathway analyses for these genes that highly correlated with the proposed fourteen lncRNAs, as well as the differentially expressed genes (DEGs) derived from the high-risk vs. low-risk groups, and the recurrence vs. non-recurrence groups, respectively. Notably, these results were consistent, and these genes were mostly enriched in the transcription factors, G protein-coupled receptors, MAPK signaling pathways, which were proved significantly associated with tumor progression and drug resistance.

**Conclusions:**

Our results suggested that the fourteen-lncRNA-based RFS predicting signature is an independent indicator for BC patients. Further prospective studies with more samples are needed to verify our findings.

## Background

Bladder cancer (BC) is known as the ninth most frequent malignancy around the world. Because of high rate of recurrence, it has been one of the most expensive solid cancer once the patients seed for continued surveillance [1]. Further, the high recurrence rate of BC is partly owning to the insufficient number of prognosis-related biomarkers. Thus, to find out more favorable biomarkers for early detection and prognosis prediction of bladder cancer is becoming more critical.

The long non-coding RNAs (lncRNAs) are characterized as RNA transcripts > 200 bases, but they cannot translate into proteins [2, 3]. Currently, plenty of studies have indicated that lncRNAs participate in diverse biological processes, such as cell proliferation [4], differentiation [5], chromatin modification, and so on [6]. The lncRNAs have been revealed playing critical roles in tumorigenesis and progression. Referring to previous studies, a series of lncRNAs, such as MALAT1, TUG1, H19, played a critical role in predicting the prognosis, the risk of metastasis [7–11], as well as drug treatment resistance [12]. Mechanisms studies found the function of lncRNA is partly depended on its location. For example, the lncRNA-HOTAIR, located in the nuclear, regulates the gene expression through recruiting the chromatin modifiers on the promoter region of transcriptional factors, enhancing their transcriptional activities. For these lncRNAs located in the cytoplasm, regulates the protein expression mostly through post-transcription, such as influencing the protein stability.

Besides, the lncRNAs could also serve as competing endogenous RNAs to influence the expression of targeted genes by competing with the microRNAs (miRNAs). In addition to the functional role of lncRNAs, many other studies have highlighted their role in predicting the prognosis of cancer patients. For example, Yang et al. [13] identified a six-lncRNA-based signature, which can predict the recurrence risk of ovarian cancer. In Song et al.’s [14] work, they established a lncRNA-based signature that provides the prognostic value for outcomes of gastric cancer patients. Similar studies were also conducted for thyroid papillary carcinoma [15], pancreatic cancer [16] and esophageal squamous cell carcinoma [17], and revealed satisfied outcomes. Although few studies have tried to construct gene-based signature for bladder cancer patients, there is no study specifically focus on muscle-invasive BC patients, who mostly have poor survival outcome than these patients with in situ tumors.

Here, we performed a systematic screening of lncRNAs that related to the recurrence-free survival (RFS) of muscle-invasive BC patients, and established a fourteen-lncRNA-based signature. Our study provides the tool for clinicians to develop the personalized medicine for muscle-invasive BC patients.

## Methods

### Bladder cancer datasets and patient information

Clinical information, particularly for RFS (status and follow-up time), and the RNA expression profile for lncRNAs in invasive BC tissues were directly download from TCGA database (https://cancergenome.nih.gov/). A total of 320 patients were diagnosed as muscle-invasive BC, and then they were divided into the training and validating groups (224 vs. 96), randomly.

### Signature construction and risk stratification

The lncRNAs were subjected to the univariable Cox regression analysis to find out lncRNAs that correlated with the RFS of muscle-invasive BC patients. Then, the LASSO Cox regression analysis was conducted to establish the risk signature based on these RFS-related lncRNA candidates. The risk value of each patient was depended on the expression of lncRNAs, and their matched co-efficient. The formula of our signature was presented as below:

$$ Risk\ score={\beta}_{\mathrm{lncRNA}1}\times {expr}_{\mathrm{lncRNA}1}+{\beta}_{\mathrm{lncRNA}2}\times {expr}_{\mathrm{lncRNA}2}+\dots +{\beta}_{\mathrm{lncRNA}\mathrm{n}}\times {\beta}_{\mathrm{lncRNA}\mathrm{n}} $$

The patients in both the training and validation cohorts were ranked by the risk scores, and then they were classified into high- or low-risk group according to the risk score of each sample (high-risk: risk score > 0; low-risk: risk score < 0).

### Kaplan-Meier (K-M) and receiving operating curve (ROC)

The K-M analysis was applied to find out the RFS difference between the low- and high-risk groups, and the ROC curve was used to determine the prognostic value of our signature in both the training and validation cohorts. Hence, the multivariate analyses were executed to determine the independent effect of our signature with clinicopathological features (such as sex, age, tumor grade and tumor stage). All these analyses were conducted based on R software (version 3.5.2) with the following R packages: ‘glmnet’, ‘survivalROC’ and ‘ggplot2’. Besides, the *P*-value less than 0.05 was considered as statistically significant.

### Differential expression and functional analysis

Differential expression analysis was performed by DESeq2 [18], and differentially expressed genes (DEGs) were defined as fold change more than 1 and adjusted *p*-value less than 0.05. Functional categories of genes were analyzed using Proteomap (https://www.proteomaps.net/).

## Results

### Signature establishment

**Fig. 1:**
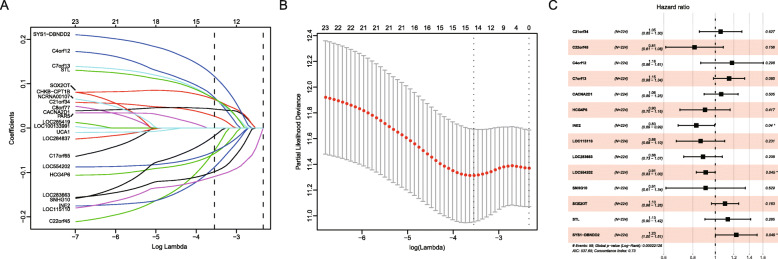
Used the LASSO Cox regression test to construct the lncRNA-based recurrence-free survival predicting classifier. **a**. LASSO coefficient profiles of the fourteen features for RFS. **b**. Tuning parameter (lamda) selection in the LASSO model used 10-fold cross-validation via minimum criteria for RFS. **c**. Forest plot showing multivariate Cox regression analysis of the effect of different lncRNAs on patient RFS

The muscle-invasive BC patients (*n* = 320) were divided into the training cohort (*n* = 224) and a validation cohort (*n* = 96), randomly. Subsequently, the univariate Cox regression test was executed to verify the RFS-related lncRNAs (Fig. S1, *P*-value less than 0.05). The predicting signature was constructed based on the training cohort with the LASSO method. As a result, the formula of the signature was defined as below (Fig. 1): Risk score = (0.052146 × expression value of C21orf34) - (0.20576 × expression value of C22orf45) + (0.164629 × expression value of C4orf12) + (0.136011 × expression value of C7orf13) + (0.057299 × expression value of CACNA2D1) - (0.10285 × expression value of HCG4P6) - (0.18816 × expression value of INE2) - (0.14616 × expression value of LOC115110) - (0.12243 × expression value of LOC283663) - (0.09584 × expression value of LOC554202) - (0.09684 × expression value of SNHG10) + (0.095918 × expression value of SOX2OT) + (0.123121 × expression value of STL) + (0.207295 × expression value of SYS1-DBNDD2). Referring to the formula, the risk score for the patients in both the training and validation cohorts was generated. Patients were ranked according to their risk scores, and referring to the median risk score of the training cohort as the cutoff point, these patients were assigned into low- and high-risk groups.

### Survival and ROC analyses

**Fig. 2:**
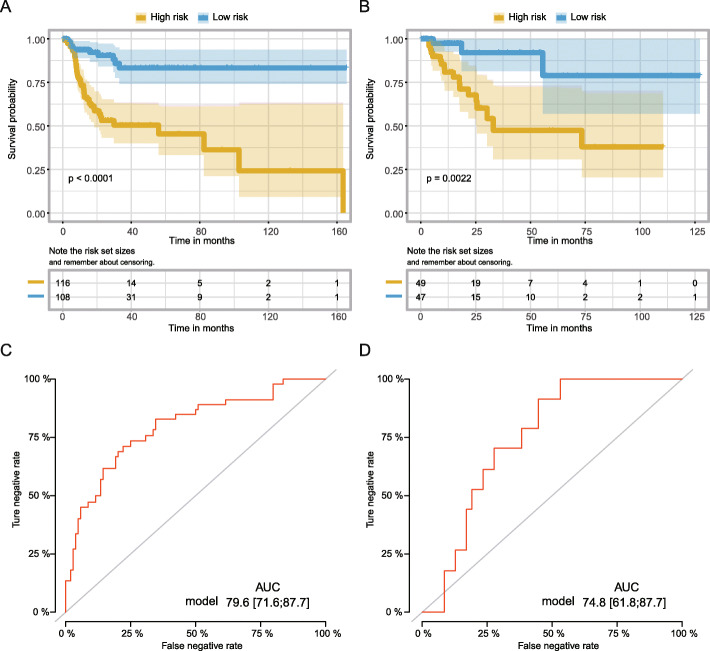
Fourteen-lncRNA-based RFS predicting classifier performance in MIBC. **a** and **b**. Kaplan-Meier curve of the low- and high-risk groups verified by the fourteen-lncRNA-based overall survival predicting classifier in the training and validation set, respectively. **c** and **d**. ROC curve of the low- and high-risk sets verified by the fourteen-lncRNA-based recurrence-free survival predicting classifier in the training and validation set, respectively

We managed the K-M analysis with the log-rank test to compare the difference in recurrence rate between the low- and high-risk groups. As shown in Fig. 2a, we found that the patients with the high-risk scores tended to experience a higher recurrence rate than for those with low-risk scores in the training cohort (*P* < 0.0001). The similar results also presented in the validation set (*P* = 0.0022; Fig. 2b). Furthermore, the ROC curves were applied to determine the predictive values of our signature. The results showed that the AUC value of the training cohort was 0.796 (95%CI: 71.6–87.7), while it was 0.748 (95%CI, 61.8–87.7) in the validation cohort (Fig. 2c and d), indicating that our signature had a moderate predictive accuracy and reliability in evaluating the prognosis for muscle-invasive BC patients.

### Multivariate analyses

**Fig. 3:**
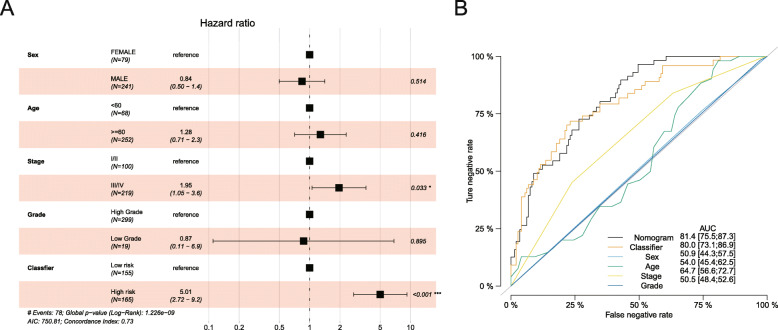
The comparison between the fourteen-lncRNA-based RFS classifier and clinicopathological features in all MIBC samples. **a**. Univariate Cox proportional-hazards regression analysis results of fourteen-lncRNA-based classifier and clinicopathological features, respectively. **b**. ROC curve of low- and high-risk sets verified by the fourteen-lncRNA-based classifier and clinicopathological features, respectively

We performed the multivariable Cox regression analysis to adjust the clinical variables (age, sex, tumor stage and grade), and the results showed our fourteen-lncRNA-based RFS and tumor stage (stage III + IV) remained to be the independent prognostic factors for muscle-invasive BC patients’ RFS in the overall dataset (Fig. 3a). The HR (HR = 5.01, 95%CI: 2.72–9.2) for the integrated lncRNA signature was greater than tumor stage (HR = 1.95, 95%CI: 1.05–3.6), and it implied that the signature had superior performance compared with the tumor stage.

Besides, we also compared our classifier with other clinicopathological features (age, sex, tumor grade, and tumor stage). We found the signature showed a high AUC value of 0.80, which was better than any of the other features (Fig. 3b). The nomogram was performed to determine the synthesis effects by combining our signature with clinicopathological features, and the results indicated that the nomogram had the best predicting values (AUC = 81.4, 95%CI: 75.5–87.3).

**Fig. 4:**
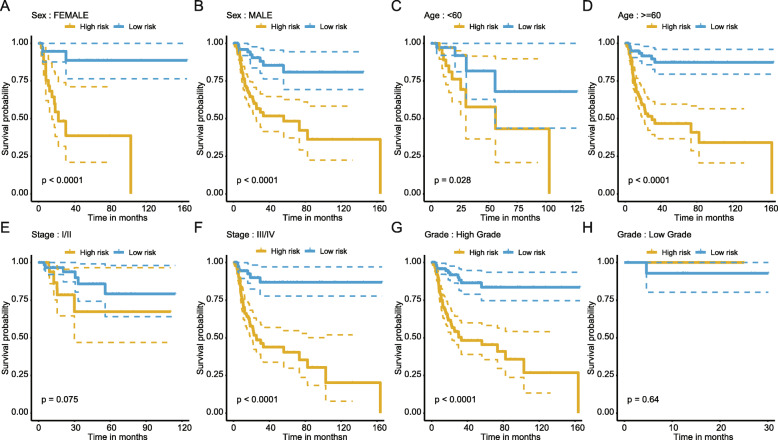
RFS classification performance of the fourteen-lncRNA-based model in subgroups of clinicopathological features. Kaplan-Meier curves show the prognostic prediction performance in subgroups of sex (**a** and **b**), age (**c** and **d**), tumor stage (**e** and **f**) as well as tumor grade (**g** and **h**)

In addition, the subgroup analyses were executed referring to the clinicopathological features (age, sex, tumor grade, and tumor stage), and the results showed that the fourteen-lncRNA-based RFS signature still could classify the risk stratification in spite of age (< 60, and > 60), sex (male, and female) (Fig. 4a-d). Further, our signature was also effective for these patients with higher stage (III/IV; *P* < 0.0001) and grade (T3 + T4; *P* < 0.0001) (Fig. 4e-h).

### Identification of fourteen lncRNA signature associated biological pathways and processes

**Fig. 5:**
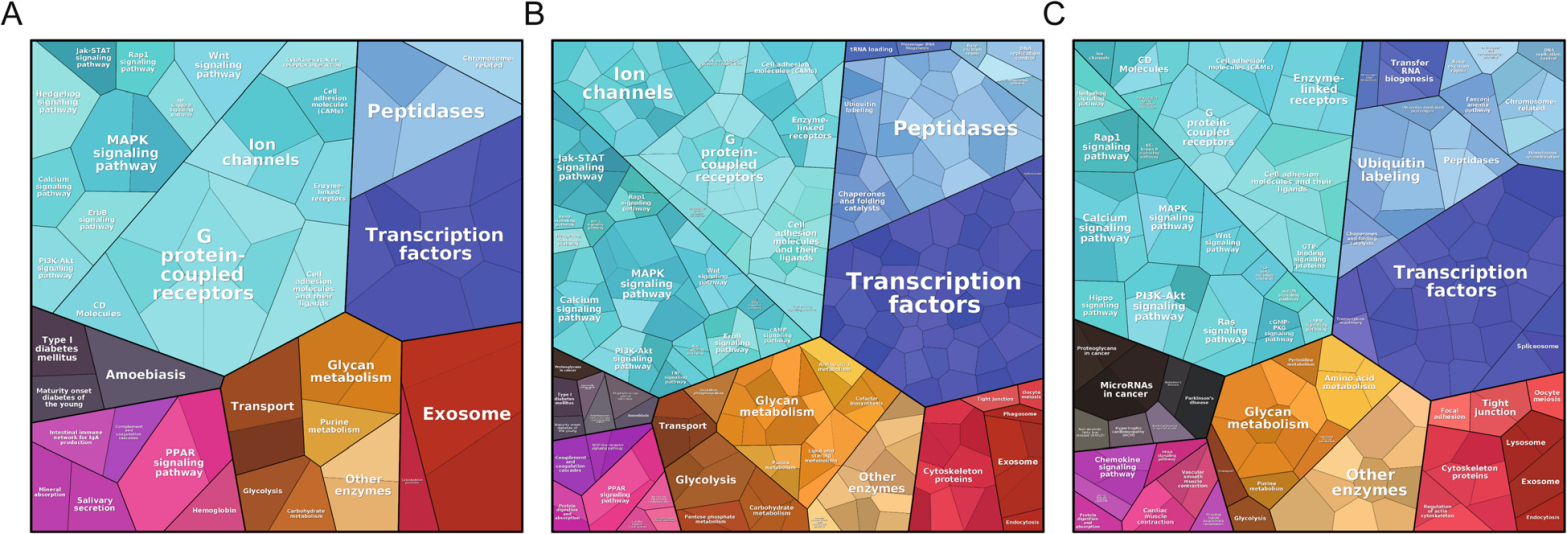
Gene functional categories showed by Proteomap. **a**. Functional category of genes which expressed highly related lncRNA markers. **b**. Functional category of genes differentially expressed between recurrent and non-recurrent MIBC samples. **c**. Functional category of genes differentially expressed between predicted high- and low-risk sample groups

To reveal the underlying mechanisms that how these fourteen lncRNAs influenced tumor progression. We extracted the mRNA expression profile from the TCGA database. For these genes highly correlated with the fourteen lncRNAs were enrolled (Pearson correlation coefficient > 0.5 or < − 0.5). Then, the proteomap pathway analysis was performed to classify their functions. The results found that these genes were mostly enriched in Transcription factors, Peptidases, Ion channels, G protein-coupled receptors, Glycan metabolism, MAPK signaling, Wnt signaling, and ErbB signaling pathways, which were proved highly associated with tumor initiation, progression and drug resistance (Fig. 5a). Notably, the DEGs were also obtained by comparing the recurrence set vs. non-recurrence set, and high-risk vs. low-risk groups, respectively, with similar results obtained (Fig. 5b and c). All these results proved the application value of our signature in predicting the RFS prognosis for muscle-invasive BC patients, and provided the potential mechanisms for how these lncRNAs influenced the BC progression.

## Discussion

The disease progression of muscle-invasive BC patients is dependent on many risk factors including phenotypes and genotypes. However, clinical criteria such as age, gender, pathological TNM stage and tumor grade may not reflect the entire biology of muscle-invasive BC. Here, we investigated the efficacy of the 14-lncRNA-based gene signature to predict the RFS of muscle-invasive BC patients. Despite previously developed gene signature-based prognostic models, it is still valuable to update new models to improve the management of muscle-invasive BC. An effective gene signature could guide patient counseling and help people to identify candidates who need more aggressive management. We demonstrated that this model has more prediction power than independent traditional clinical features. Although it is lack of novelty and function work in our study and our results require further investigation of the efficacy of the 14-lncRNA-based signature panel in patients, this panel could be extremely beneficial to identify patients at elevated risk of recurrence that may require adjuvant therapy.

We identified a set of 14 lncRNAs that showed differential expressions between high-and low-risk cancer patients included in the data sets (Fig. S2). Such differentiations signified their potential roles in carcinogenesis. Recent researches has found that lncRNA LOC554202 is significantly downregulated in bladder cancer tissues compared with adjacent noncancerous tissues, and lncRNA LOC554202 expression level in bladder cancer patients was negatively associated with advanced TNM stage [19]. SNHG10 is known to be over-expressed in hepatocellular carcinoma, and we found it facilitates hepatocarcinogenesis and metastasis [20]. SOX2 overlapping transcript mainly play crucial role in tumor initiation and/or progression as well as regulating pluripotent state of stem cells [21]. CACNA2D1 is most the most extensively investigated and validated of these markers. A retrospective study showed that positive expression of Cacna2d1 was significantly associated with advanced FIGO stage (*P* < 0.001), histological subtype (*P* = 0.017) and tumor differentiation (*P* = 0.015) [22]. Data coming from Sui et al.’s research [23] have confirmed that radio-resistance of non-small cell lung cancer induced by CaCna2D1. The roles of the rest of the lncRNA genes identified in bladder cancer remain unclear.

## Conclusion

By applying public TCGA data, we successfully built and validated a RFS prediction model of muscle-invasive BC based on a novel 14-lncRNA signature. Comparing with independent clinical features, this model has more efficiency to predict RFS of muscle-invasive BC. The model may help facilitate doctor-patient consultations, guide muscle-invasive BC treatment strategy and eventually benefit patients.

## Supplementary information

**Additional file 1: Supplementary Figure 1.** Kaplan-Meier curves show 23 lncRNAs which significantly related to recurrence-free survival of MIBC in the training data.

**Additional file 2: Supplementary Figure 2.** Expression level of the fourteen prognostic lncRNA markers in high- and low-risk MIBC groups, respectively.

## Data Availability

Not applicable.

## Abbreviations

BC: Bladder cancer
DEGs: Differentially expressed genes
FDR: False discovery rate
K-M: Kaplan-Meier
lncRNA: long-noncoding RNA
miRNAs: microRNAs
RFS: Recurrence-free survival
ROC: Receiver operating characteristic
TCGA: The Cancer Genome Atlas
