# Supplementary material for: A prognostic index based on a fourteen long non-coding RNA signature to predict the recurrence-free survival for muscle-invasive bladder cancer patients
Source: BMC Med Inform Decis Mak. 2020 Jul 9;20(Suppl 3):136. doi: 10.1186/s12911-020-1115-2 (PMC7346316; doi:10.1186/s12911-020-1115-2)
Supplement: Supplementary file 1 — Additional file 1: Supplementary Figure 1. Kaplan-Meier curves show 23 lncRNAs which significantly related to recurrence-free survival of MIBC in the training data. [file 12911_2020_1115_MOESM1_ESM.pdf]

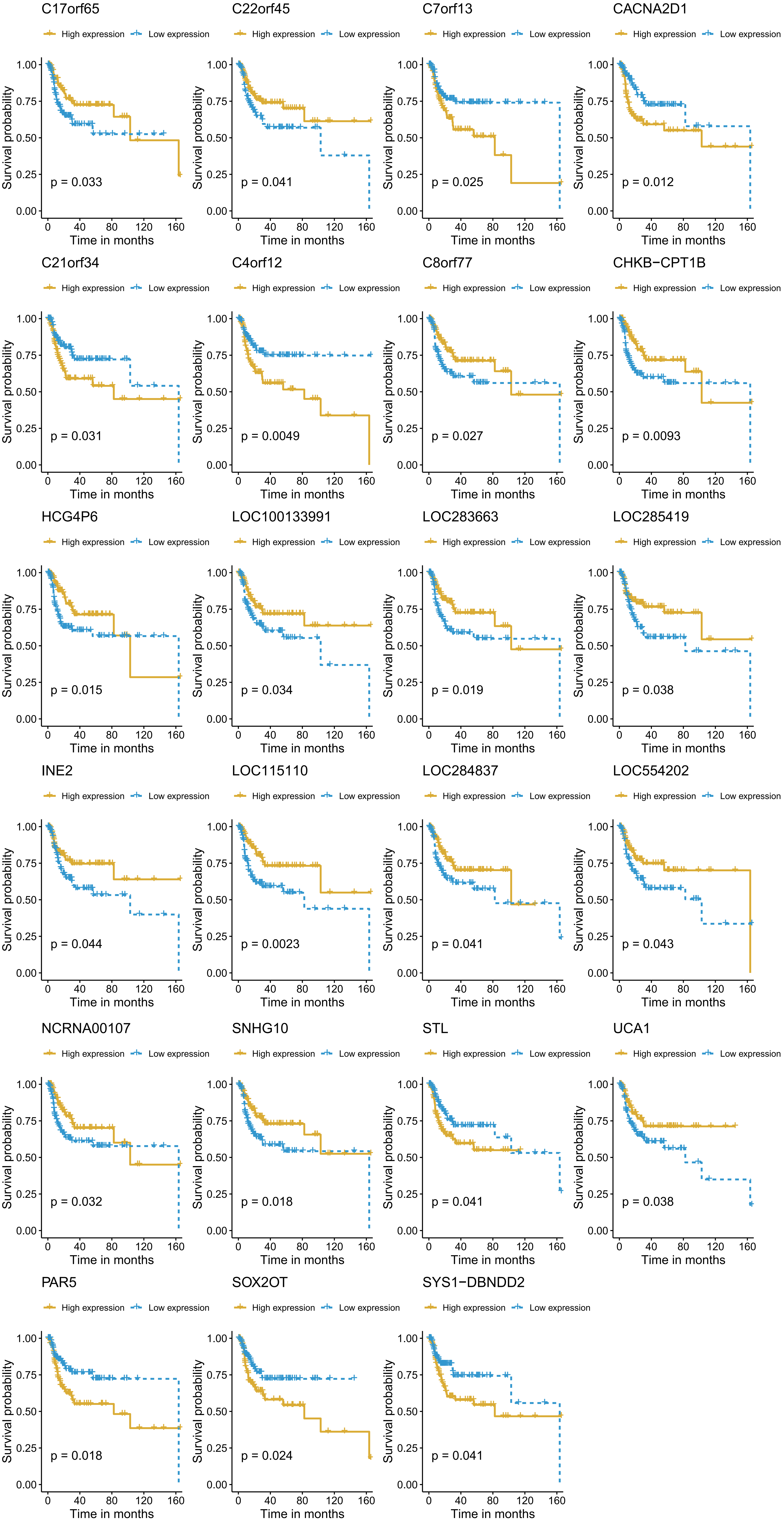
C17orf65
High expression
Low expression
Survival probability
1.00
0.75
0.50
0.25
0.00
0
40
80
120
160
Time in months
p = 0.033
C22orf45
p = 0.041
C7orf13
p = 0.025
CACNA2D1
p = 0.012
C21orf34
p = 0.031
C4orf12
p = 0.0049
C8orf77
p = 0.027
CHKB−CPT1B
p = 0.0093
HCG4P6
p = 0.015
LOC100133991
p = 0.034
LOC283663
p = 0.019
LOC285419
p = 0.038
INE2
p = 0.044
LOC115110
p = 0.0023
LOC284837
p = 0.041
LOC554202
p = 0.043
NCRNA00107
p = 0.032
SNHG10
p = 0.018
STL
p = 0.041
UCA1
p = 0.038
PAR5
p = 0.018
SOX2OT
p = 0.024
SYS1−DBNDD2
p = 0.041
