# Supplementary figures and images for: A prognostic index based on a fourteen long non-coding RNA signature to predict the recurrence-free survival for muscle-invasive bladder cancer patients
Source: BMC Med Inform Decis Mak. 2020 Jul 9;20(Suppl 3):136. doi: 10.1186/s12911-020-1115-2 (PMC7346316; doi:10.1186/s12911-020-1115-2)

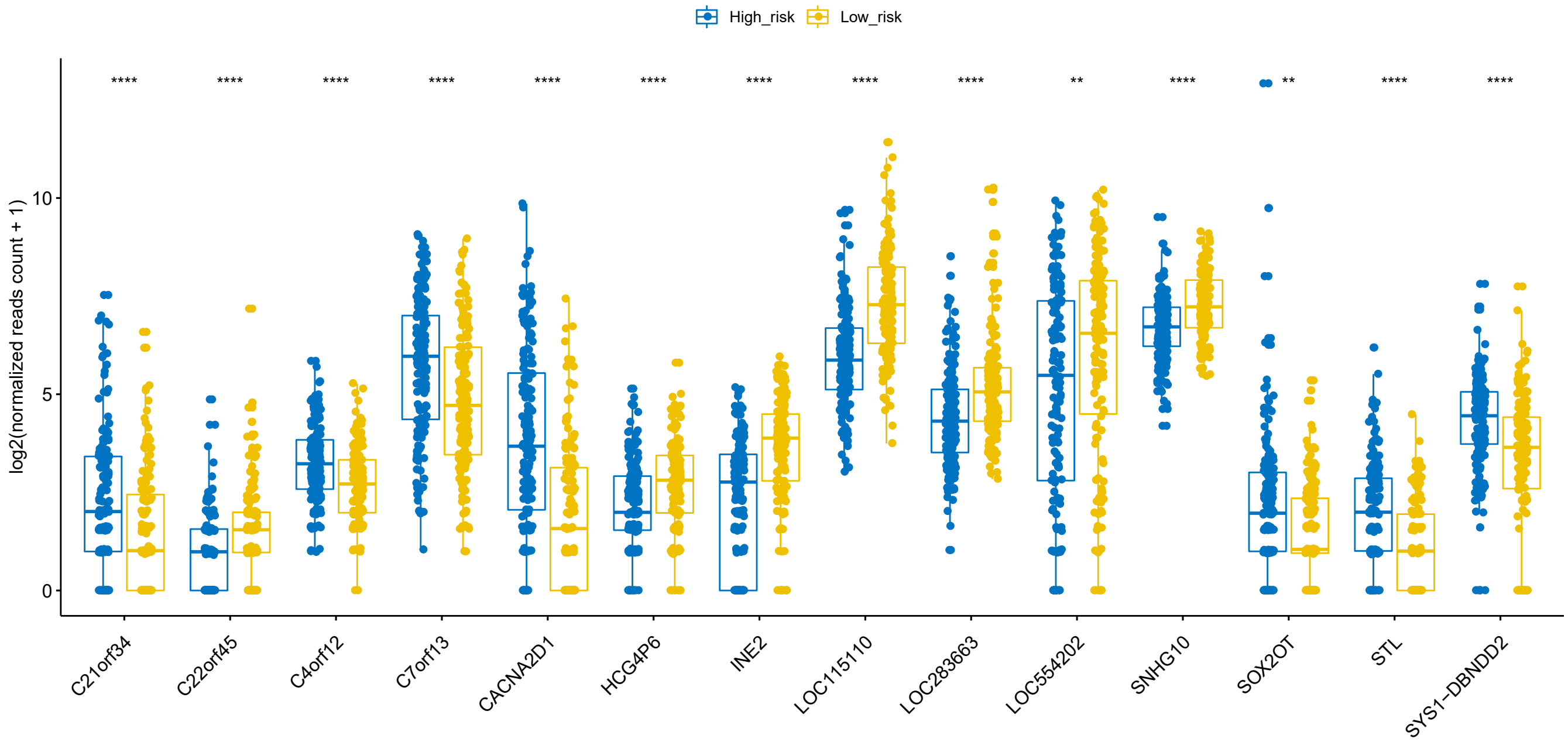

Supplement: Supplementary file 2 — Additional file 2: Supplementary Figure 2. Expression level of the fourteen prognostic lncRNA markers in high- and low-risk MIBC groups, respectively. [file 12911_2020_1115_MOESM2_ESM.pdf]
